# The natural compound chebulagic acid inhibits vascular endothelial growth factor A mediated regulation of endothelial cell functions

**DOI:** 10.1038/srep09642

**Published:** 2015-04-10

**Authors:** Kai Lu, Sujit Basu

**Affiliations:** 1 Department of Pathology, Ohio State University, Columbus, Ohio 43210, United States of America; 2 Division of Medical Oncology, Department of Internal Medicine, Ohio State University, Columbus, Ohio 43210, United States of America

## Abstract

Vascular endothelial growth factor A (VEGFA) plays an important role in tumour angiogenesis and its angiogenic action is mainly mediated through its VEGF receptor 2 (VEGFR-2). Therefore drugs targeting VEGFA/VEGFR-2 are being presently used in the clinics for treatment of several types of solid malignant tumours. We here in report that low dose of chebulagic acid (CA), a hydrolysable tannin found in myrobalan fruits can inhibit VEGFA induced vascular permeability, endothelial cell proliferation, migration, tube formation and thereby, angiogenesis by suppressing VEGFR-2 phosphorylation. CA may thus be an effective and useful natural inhibitor of VEGFA mediated angiogenesis.

VEGFA induced new blood vessel formation is essential for promotion, progression and metastasis of solid tumours^1^,^2^. VEGFA by acting mainly via its VEGFR-2 present in the endothelial cells promotes angiogenesis by inducing microvascular permeability, endothelial cell proliferation, migration and tube formation^1^,^2^. Accordingly, several anti-angiogenic agents are being presently used in the clinics targeting this cytokine and VEGFR-2 for the treatment of several types of solid tumours^3^,^4^. However, these anti-angiogenic drugs are very expensive and it is therefore prudent to identify newer, inexpensive and effective anti-angiogenic molecules^5^.

We have very recently demonstrated that Triphala (THL), a mixture of three myrobalan fruits can significantly inhibit VEGFA induced angiogenesis^6^,^7^. Previous reports have indicated that in addition to gallic acid and ellagic acid, chebulagic acid (CA), benzopyran tannin is also a major constituent of THL^8^,^9^. Since plasma levels and bioavailability of gallic acid and ellagic acid following oral ingestion are poor^10^,^11^, therefore it is possible that other bioactive constituents of THL may be responsible for the anti-VEGFA actions of this mixture^7^. This study thus investigated the effects of CA at a concentration present in THL on VEGFA mediated regulation of proangiogenic endothelial cells functions.

## Results and Discussion

### Plasma concentration of CA in mice after oral feeding of THL

**Figure 1:**
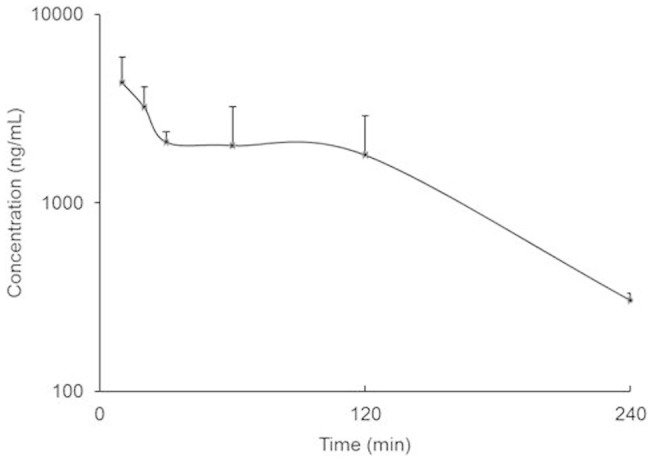
The plasma concentration of chebulagic acid (CA). The plasma concentration-time profile of chebulagic acid in mice after oral ingestion of a single dose (100 mg/kg) of Triphala (n = 3). The error bars represent SEM.

Since our previous report indicated significant inhibition of VEGFA mediated angiogenesis by THL and because CA present in the THL may have anti-VEGFA effects, we therefore at first investigated the plasma level of CA after gavaging mice with THL^7^,^8^,^9^. Our LC-MS/MS data indicated that the concentrations of CA in plasma were 4341.67 ng/ml (~5 μM) at 10 min, 2098.33 ng/ml (~2 μM) at 30 min and 1795.00 ng/ml (~1 μM) at 2 hr after oral feeding of these animals with a single non-toxic dose of 100 mg/kg of THL containing 22.2 mg/kg of CA (Figure 1).

### Cytotoxic effects of CA on endothelial cells

**Figure 2:**
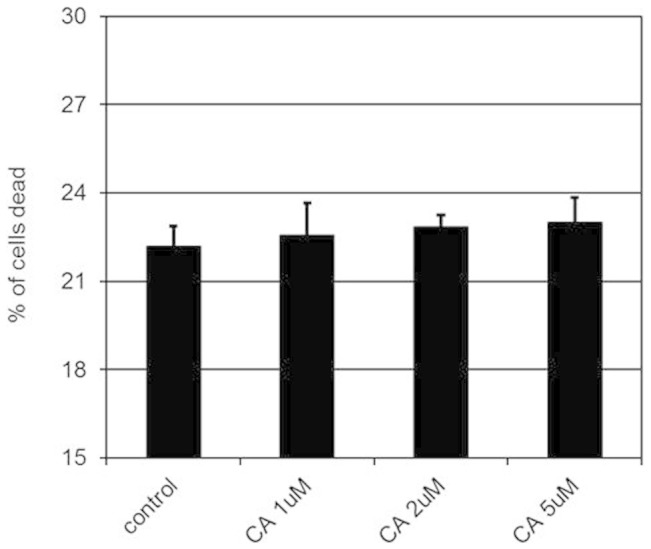
The effects of chebulagic acid (CA) on the viability of endothelial cells. The cytotoxic effects of 1, 2 and 5 μM of chebulagic acid (CA) on human umbilical vein cells (HUVEC) (*, *p <* 0.05). The error bars represent SEM. The data represent six separate experiments.

Thereafter, the effects of 1, 2 and 5 μM of CA on the viability of human umbilical vein endothelial cells (HUVEC) were determined because these plasma concentrations of CA reached when mice were gavaged with a single non toxic VEGFA inhibitory dose (100 mg/kg) of THL (Figure 1). Our results indicated that 1, 2 and 5 μM of CA had no effects on cell viability (Figure 2). Therefore 1, 2 and 5 μM of CA were selected for our *in vitro* experiments.

### Effects of CA on endothelial cell functions

**Figure 3:**
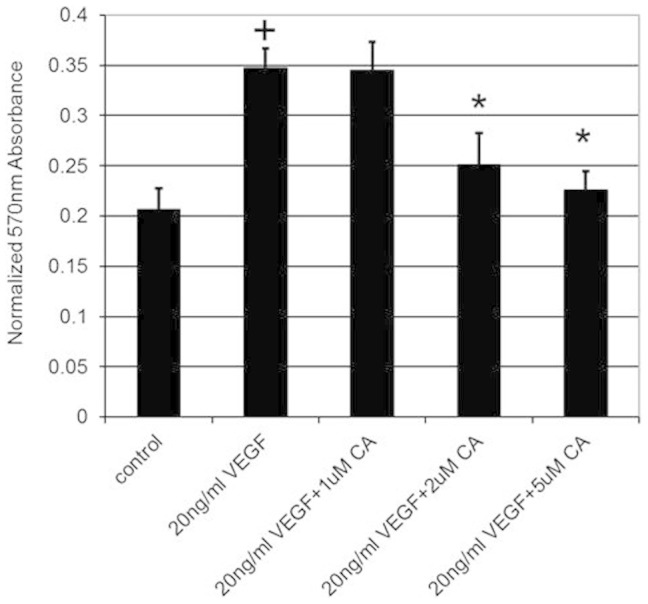
The effects of various concentrations of chebulagic acid (CA) on vascular endothelial growth factor A (VEGFA) mediated proliferation of endothelial cells. VEGFA stimulated proliferation of human umbilical vein cells (HUVEC) when compared to untreated control (+, *p* < 0.05). However, this stimulatory effect was inhibited when these cells were also treated with 2 and 5 μM of CA (*, *p* < 0.05). The error bars represent SEM. The data represent six separate experiments.

**Figure 4:**
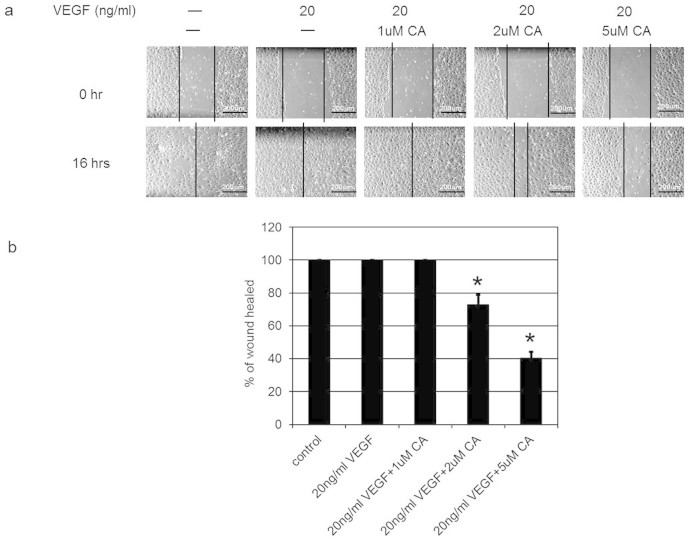
The effects of chebulagic acid (CA) on vascular endothelial growth factor A (VEGFA) induced migration of human umbilical vein cells (HUVEC). Phase-contrast photomicrographs demonstrated VEGFA induced complete wound closure or healing at 16 hr. On the contrary, this effect of VEGFA was abrogated when these cells were treated with 2 and 5 μM of CA. The wound healing was calculated as the distance covered by the cells in relation to the initial wound distance at 0 hr and is expressed as the percentage of initial distance at 0 hr. (*, *P* < 0.05). The error bars represent SEM. Scale bars in a, 200 um. The data represent six separate experiments.

**Figure 5:**
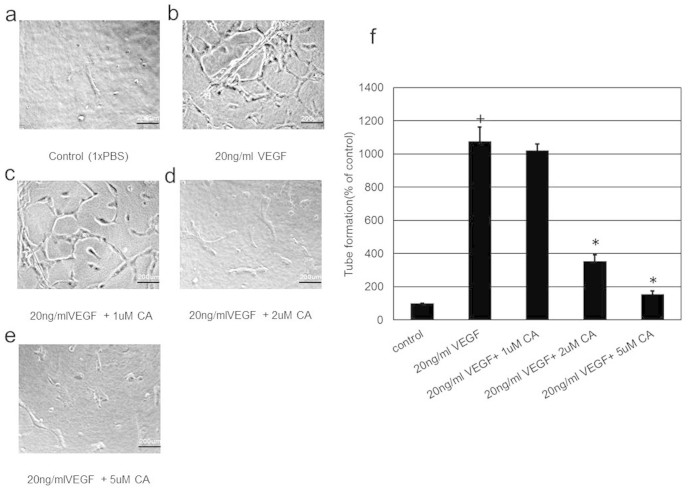
The effects of chebulagic acid (CA) on vascular endothelial growth factor A (VEGFA) mediated tube formation in endothelial cells. VEGFA stimulated tube formation in human umbilical vein cells (HUVEC) when compared to untreated control (+, p < 0.05). On the contrary, treatment with 2 and 5 μM of CA inhibited VEGFA induced endothelial cell tube formation (*, *p* < 0.05). Scale bars in (a), (b), (c), (d) and (e), 200 um. The data represent six separate experiments.

**Figure 6:**
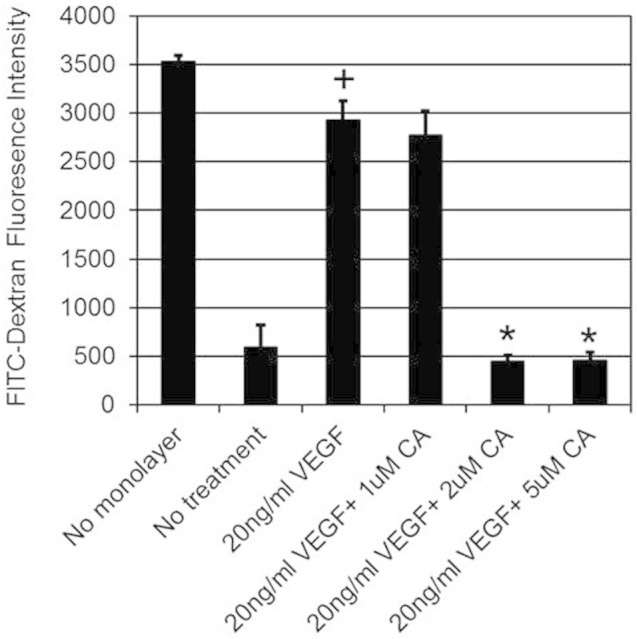
The effects of chebulagic acid (CA) on vascular endothelial growth factor A (VEGFA) induced permeability in endothelial cells. In comparison to untreated control, VEGFA induced significant permeability in human umbilical vein cells (HUVEC) (+, *p* < 0.05). On the contrary, treatment with 2 or 5 μM of CA significantly inhibited VEGFA stimulated permeability in HUVEC (*, *p* < 0.05). The data represent six separate experiments.

**Figure 7:**
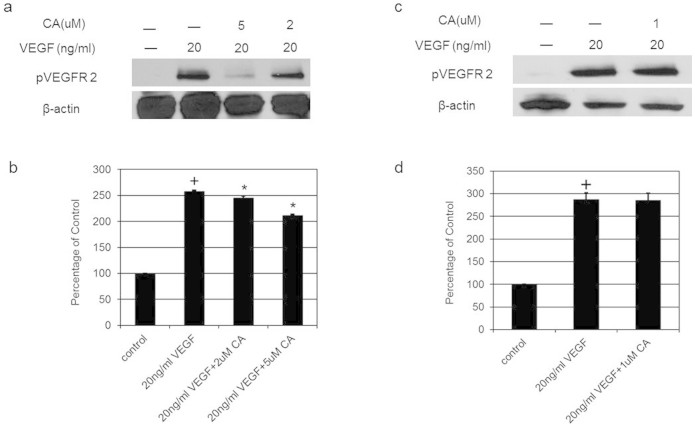
The effects of chebulagic acid (CA) on vascular endothelial growth factor A (VEGFA) induced phosphorylation of VEGF receptor 2 (VEGFR-2). Western blot analysis shows that in comparison to untreated control, VEGFA induced significant VEGFR-2 phosphorylation (+, *p* < 0.05). However, there was also a significant inhibition of VEGFA induced phosphorylation of VEGFR2 after treatment with 2 or 5 μM of CA (*, *p* < 0.05). The blot was re-probed with an antibody to β actin for comparison of equal protein load. Cropped gel images have been used in this figure and the gels were run under the same experimental conditions. The data represent six separate experiments. Full-length blots are presented in Supplementary Figure S1.

Since VEGFA induces angiogenesis by stimulating endothelial cell permeability, proliferation, migration and tube formation^1^,^2^, we thus investigated the effects of plasma levels (1, 2 and 5 μM) of CA detected in mice following THL intake on these VEGFA mediated endothelial cell functions. We observed that 2 and 5 μM of CA significantly inhibited VEGFA (20 ng/ml) induced endothelial cell proliferation (Figure 3), migration (Figure 4a and 4b) and tube formation (Figure 5a, 5b, 5c, 5d, 5e and 5f). In addition, 2 and 5 μM of CA also significantly inhibited VEGFA mediated endothelial cell permeability *in vitro* (Figure 6). On the contrary, no such effects were observed when these cells were treated only with CA (data not shown). In addition, we observed that 2 and 5 μM of CA could significantly inhibit VEGFA induced VEGFR-2 phosphorylation in these cells (Figure 7a and 7b), thereby suggesting the molecular mechanism of VEGFA inhibitory actions of this natural compound. However, 1 μM of CA had no such significant effects on VEGFA mediated activation of VEGFR-2 (Figure 7c and 7d).

### Effects of CA on angiogenesis in chick chorioallantoic membrane (CAM) assay

**Figure 8:**
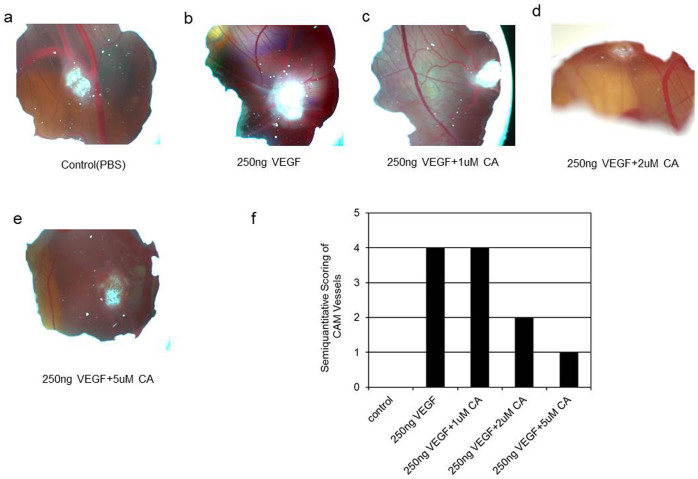
The effects of chebulagic acid (CA) on vascular endothelial growth factorA (VEGFA) induced new blood formation or angiogenesis in the CAM assay. (a, f) Phosphate buffered saline (PBS) used as control did not induce blood vessel formation. (b, f) On the contrary, VEGFA stimulated new blood vessel formation. (c, f) 1 μM of CA did not inhibit VEGFA induced angiogenesis. (d, e, f) 2 or 5 μM of CA inhibited VEGFA induced new blood vessel formation. The photograph represents six separate experiments.

Furthermore as our *in vitro* data indicated that CA could significantly inhibit the pro-angiogenic actions of VEGFA (Figure 3, 4, 5, 6, 7), we next examined the effects of CA (1, 2 and 5 μM) on VEGFA induced angiogenesis using CAM assay^7^,^12^,^13^. New blood vessel formation or angiogenesis was not detected on treatment with the vehicle (phosphate buffered saline solution) (Figure 8a and 8f). In contrast, prominent angiogenesis was seen on treatment with 250 ng of VEGFA (Figure 8b and 8f). Furthermore, although 1 μM of CA had no prominent effects, there was significant inhibition of VEGFA mediated angiogenesis after treatment with 2 and 5 μM of CA (Figure 8c, 8d, 8e and 8f). Importantly, CA alone had no effects on angiogenesis (data not shown). These observations were made on Day 4 after treatment with CA.

In conclusion, this study demonstrates that CA by suppressing VEGFA mediated activation of VEGFR-2 inhibits the pro-angiogenic actions of this cytokine on endothelial cells. Because angiogenesis plays an important role in many pathological conditions such as malignant tumours, rheumatoid arthritis and diabetic retinopathy, therefore this natural molecule may be used as an effective anti-angiogenic agent for the treatment of these diseases in future^14^.

## Methods

### Reagents

Triphala and more than 90% pure CA were purchased from Dabur, New Delhi, India and Natural Remedies, Bangalore, India respectively. The recombinant human VEGFA was procured from R&D systems, MN, USA. The CA and other solutions used for the experiments were free from endotoxin as determined by gel-clot limulus amebocyte lysate assay (Charles River, MA, USA)^15^. All other chemicals were from Sigma, MO, USA.

### Detection of plasma CA in mice

All animal experiments were undertaken following the approved protocol of Institutional Animal Care and Use Committee of the Ohio State University. 6–8 wks old, C57BL/6 male mice were at first gavaged with a single non-toxic dose (100 mg/kg) of THL^7^. Blood was then collected by cardiac puncture at different time intervals followed by separation of plasma. The level of CA in plasma was finally determined by LC-MS/MS^7^.

### Endothelial cell culture

HUVEC were cultured in endothelial basal medium (EBM) supplemented with growth factors and 2% FBS (Lonza, CA, USA). All HUVEC experiments unless specified were undertaken in 24 hrs serum and growth factor starved cells^7^,^16^,^17^.

### *In vitro* toxicity assay

In order to determine the cytotoxicity of CA, trypan blue dye exclusion test was done to examine the viability of the cells. 2 × 10^4^ endothelial cells were at first treated with 1, 2 and 5 μM of CA. 200 μl of 0.4% w/v trypan blue (Sigma-Aldrich, MO, USA) was then added to these cells followed by counting of the stained cells^12^.

### Proliferation assay

5 × 10^3^ HUVEC were either untreated or treated with VEGFA (20 ng/ml) or VEGFA (20 ng/ml) + CA (1, 2 and 5 μM) for 24 hr. Prestoblue™ Cell Viability reagent (Invitrogen, NY, USA) was used as per instructions of the manufacturer to detect proliferation of these cells^7^,^18^.

### Wound healing assay

After wounding the HUVEC monolayers with a 200 μl pipette, the effects of CA on VEGFA mediated migration were determined in both untreated cells or cells treated with VEGFA (20 ng/ml) or VEGFA (20 ng/ml) + CA (1, 2 and 5 μM). The closure of wound was determined at 16 hr by the distance covered by these cells in relation to the initial distance. The results were expressed as percentage of wound healed^7^,^17^.

### Endothelial cell tube formation assay

*In vitro* angiogenesis assay kit (Millipore, CA, USA) was used as per the manufacturers instructions to determine the effect of CA on HUVEC tube formation. In brief, HUVEC treated either with phosphate buffered saline or VEGFA (20 ng/ml) or VEGFA (20 ng/ml) + CA (1, 2 and 5 μM) were seeded on extra cellular matrix to form capillary tubes. The tubular structures were manually quantified by counting the numbers of connected cells in randomly selected fields and the total tube numbers in the control group was designated as 100%^7^,^19^.

### *In vitro* endothelial cell permeability assay

This assay was undertaken as per the instructions of the manufacturer using *in vitro* vascular permeability assay kit (Millipore, CA, USA). 2 × 10^5^ HUVEC were seeded onto collagen coated inserts in 24 well plates. The respective inserts containing HUVEC were then pre-treated with 1, 2 and 5 μM of CA for 24 hr. Thereafter, FITC-Dextran containing phenol free medium containing 20 ng/ml of VEGFA was added and the fluorescence was measured at 485 nm/535 nm^7^,^17^.

### Western Blot

Rabbit monoclonal antibody against phospho VEGFR-2 purchased from Cell Signaling, MA, USA was used to detect VEGFR-2 phosphorylation. The protein loads were normalized using antibody for β actin (Sigma, MO, USA) and the antibody reactive bands were quantified by densitometry^7^,^20^,^21^.

### Chick chorioallantoic membrane (CAM) assay

Semi-quantitative CAM assay was utilized to determine the effects of CA on VEGFA stimulated new blood vessel formation or angiogenesis. At first, windows were created on the shells of Leghorn chicken eggs (Ohio State University Farms, Columbus, USA) at day 3 of fertilization to expose the CAM. 1-mm^3^ sterilized gelatin sponge (Pfizer, MI, USA) soaked either phosphate buffered saline (PBS) (control), or VEGFA (250 ng) or VEGFA (250 ng) + CA (1, 2 and 5 μM) was then aseptically inserted onto the CAM on day 8. Formation of new blood vessel formation was scored on day 12 as negative (0); change in vessel architecture (0.5); partial spoke of a wheel (1/3 of circumference exhibited directional angiogenesis) (1); spoke of a wheel (2); strong and fully spoke of a wheel (≥3)^7^,^12^,^13^. Nikon D70 camera with AF Micro Nikkor 105 mm lens was used to take the photographs^7^.

### Statistical analysis

ANOVA and unpaired Student's t test or Dunn's multiple comparison tests were used to analyze the differences among the groups. *P* < 0.05 was considered to be significant^16^,^20^.

## Supplementary Material

Supplementary Information Full Length Blot of Figure 7
