# Supplementary material for: The natural compound chebulagic acid inhibits vascular endothelial growth factor A mediated regulation of endothelial cell functions
Source: Sci Rep. 2015 Apr 10;5:9642. doi: 10.1038/srep09642 (PMC4819393; doi:10.1038/srep09642)
Supplement: Supplementary Information — Full Length Blot of Figure 7 [file srep09642-s1.pdf]

The natural compound chebulagic acid inhibits vascular endothelial growth factor-A mediated regulation of endothelial cell functions

Kai Lu[1] & Sujit Basu [1, 2*]

[1]Department of Pathology and [2]Division of Medical Oncology, Department of Internal Medicine, Ohio State University, Columbus, Ohio 43210, United States of America

*Correspondence and requests for materials should be addressed to Sujit Basu (sujit.basu@osumc.edu)

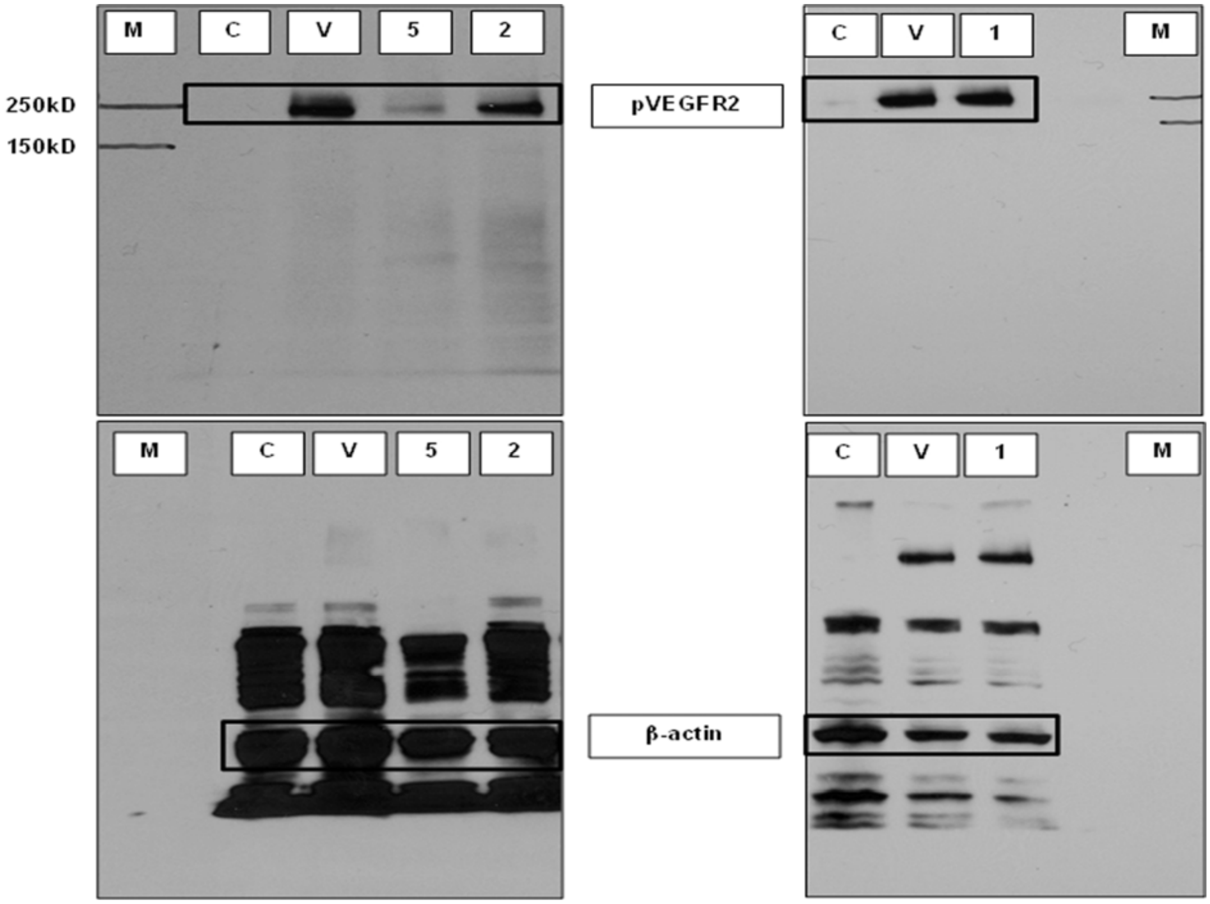

Figure S1. Full length blots of Figure 7. M: marker; C: untreated control; V: 20 ng/ml of VEGFA treated; 1: treated with 20 ng/ml of VEGFA + 1μM chebulagic acid; 2: treated with 20 ng/ml VEGFA + 2μM chebulagic acid and 5: treated with 20 ng/ml of VEGFA + 5μM chebulagic acid.
